# Understanding of Stigmatization and Death Amid COVID-19 in India: A Sociological Exploration

**DOI:** 10.1177/00302228211008753

**Published:** 2021-04-22

**Authors:** Sarita Kumari

**Affiliations:** Department of Sociology, Delhi School of Economics, University of Delhi, New Delhi, India

**Keywords:** coronavirus, stigmatization, health seeking behaviour, death, crematorium and bereaved families

## Abstract

The unanticipated outbreak of Coronavirus had proven detrimental to human existence. It had created waves of panic, anxiety, and fear among people hence facilitating stigmatization toward an infected person. This stigmatization further influences patients health-seeking behaviour due to the trust deficit in the public health system. The virus had placed the world in an impotent situation as people helplessly watched their loved ones pass away in the absence of effective treatment. Dead bodies are denied a dignified death due to mandatory guidelines prescribed by countries to control the pandemic. This article attempts to understand the process of stigmatization of Coronavirus and its mechanism of influencing the health-seeking behaviour of people. Moreover, the way this stigmatization, accompanied by fear and anxiety, led to the denial of having a dignified death in India.

## Introduction

The Coronavirus cases are surging at an unprecedented scale across the world. It results in escalating death rates with a much higher pace as many infected dead bodies are left isolated, lying unattended in houses, street and mortuary (Gray, 2020; Horowitz & Emma, 2020). These extreme conditions had arisen as resultants of specific guidelines and instructions planned in a haste manner by authorities, for instance, an immediate ban on funeral ceremonies in some countries. The world is forced to witness this historical period by remaining meek spectators of the devastations caused by the pandemic. It had created waves of loss, grief and fear among people. Maddrell (2020) had rightly pointed out that as ‘if those frequently exposed to the virus are at risk of ‘acute viral loads’, so those who are exposed to a high frequency of traumatic death and/or personal crises are experiencing a high ‘emotional-viral-load’ (Maddrell, 2020, p.110).

## Novel Coronavirus as Pandemic

The pandemic caused by COVID-19 is known as acute respiratory illness. Its causative agent is the SARS-Cov-2 virus that belongs to the Coronaviridae family and is enveloped with positive single-stranded RNA virus (WHO, 2019). The main transmission route of the virus is through inhalations of the respiratory droplet and its deposition on the mucosal surface. But certain research proposed that contact with contaminated bodily excretions, and the fecal-oral route can also spread it (Vidua et al., 2020). Authorities had highlighted certain crucial measures such as isolations, identifications and use of Personal Protective Equipment (PPE) to protect oneself from infection. The director of the World Health Organization (WHO) had laid stress on the significance of contact-tracing in controlling this pandemic. He mentioned that as this technique had been useful in controlling other major outbreak such as smallpox, Polio and Ebola, thus it would be helpful in controlling COVID-19 too (WHO, 2020b).

Though the virus was discovered in 2019 still, the condition of the world is petrifying. Despite excelling in technology and development even developed nations until this date had failed to provide an effective cure for this deadly virus. Several had died waiting for a cure. Situations had turned so distressing that morgues were filled with dead bodies and hospitals were overcrowded with patients. Thus, it had demonstrated the ineffectiveness of our health system in dealing with such major outbreaks. In India, hospitals denied treatment to many patients due to insufficient hospital equipment such as beds and efficient health personnel and PPE kits. Condition was so distressing that India Medical Association (IMA) had issued a red alert to medicos and medical administration safety involved in Coronavirus containment efforts (Sahay, 2020). Besides, out of pocket expenditure for the treatment exceeds one hundred thousand for ten days (M. Singh, 2020). Arrangement of such a huge fee is difficult for low-income families, and this situation points towards the failure of the government in effectively dealing with Coronavirus. Due to these extreme situations, the Supreme Court of India had intervened and ordered the government to frame a guideline on the cost of COVID treatment. It further added that no patient should be turned out of the hospital due to the high expense of COVID-19 treatment (Thomas, 2020). Besides these, numerous challenges were encountered while seeking effective treatment against Coronavirus: The first issue was getting tested for the presence of Coronavirus. Even private laboratory failed to provide a hurdle-free COVID-19 test despite charging around four thousand five hundred fees for a single test. Regardless of having 30 per cent testing labs in the private sector, only 12-18 per cent of the total numbers of samples were tested in private labs (Dey, 2020).

The second problem involves getting a timely report as the absence of this led to the denial of hospitalization for the patients. In the meantime, the patients' conditions further deteriorated and, in many cases, people had lost their lives in the ambulance or within their home due to the unavailability of the COVID-19 test report.

The third factor involves a reluctant and uncooperative attitude of people in revealing their symptoms due to the stigma attached to Covid-19, resulting in difficulties in earlier diagnosis of the disease. Another challenge emerged out due to certain technicalities involved such as after the death of the COVID patient, it was mandatory to contact the higher authorities before performing the last rites of the deceased. Further, due to constraints of crucial resources, many cases were going unreported in India.

The way stigma influenced our day-to-day life is a well-researched area but the mechanism through which stigma symbolised Coronavirus patients’ identity needed to be explored. The stigmatized identity of the COVID-19 patient results from absences of an effective treatment along with higher virulence and incidence rate associated with the virus. Thus, this article attempts to understand the process of stigmatization of Coronavirus and the way it influences the health-seeking behaviour of people. Further, some incidences highlight that dead bodies were denied a dignified death due to the fear and stigma attached to Coronavirus. For instance, lately in Bihar when a COVID patient committed suicide in a quarantine centre, the administration paid fifteen hundred rupees to a man for completing the funeral of the dead body. The person eloped with the amount and left the dead body half-burnt. Later, stray dogs were feeding on the body (Mishra, 2020). This was just one incident. Many similar cases were happening daily. Hence this article tries to understand the way this stigmatization accompanied by fear and anxiety associated with the pandemic outbreak led to a denial of having a dignified death in India.

## Stigmatization and Infectious Diseases

As the Coronavirus pandemic is escalating at a higher pace in India after affecting millions of people, society faces an existential crisis with fear and anxiety. Strong (1990) defines an epidemic outbreak as an existential threat and states that:

‘*large, fatal epidemic seems to present to social order; on the waves of fear, panic, stigma, moralizing and calls to action that seem to characterize the immediate reaction. Societies are caught up in an extraordinary emotional maelstrom which seems, at least for a time, beyond anyone’s immediate control. Moreover, since this strange state presents such an immediate threat, actual or potential, to public order, it can also powerfully influence the size, timing and shape of the social and political response in many other areas affected by the epidemic’* (p. 249).

Out of anxiety and fear, people often use ‘stigma’ to maintain avoidance from an infected person. Infectious diseases threaten the community’s ability to function effectively by tapping on its individual and spreading through those individuals during their interaction within the social system (Smith, 2007, p. 464). Thus, the society’s reactions to the novel disease and infectious agents are generally attributed to avoidance of contact and thus minimizing chances of contagion. It is often reflected in the process of quarantine at the societal level, a process which is historically deployed, such as in the case of the Human immunodeficiency virus (HIV) epidemic, followed by severe acute respiratory syndrome (SARS) and H1N1 influenza pandemic alert. Even at an individual level too the avoidance of diseases people is observed. This avoidance continues even during the post-recovery phase, also, as social exclusion has been widely observed for children who had contracted the H1N1 virus (Oaten et al., 2011).

Goffman’s (1963) pioneer work on stigma elucidates that stigmatization occurs when the evaluation of an individual results in that person being discredited. Stigma for persons leads to be ‘systematically excluded from particular sorts of social interactions because they possess a particular characteristic or are a member of a particular group’ (Kurzban & Mark.R., 2001, p. 187). Studies have already highlighted the particular characteristics of stigma relevant to understanding the Coronavirus pandemic: First, any contagious disease is highly stigmatized if it is perceived to be contracted through voluntary and avoidable behaviour. Second, intense stigma is attached to those conditions which are lethal and incurable. Third, greater stigma is associated with a condition where it poses a risk for others (Herek, 2002, p. 596). Thus, due to the fear of potential stigmatisation, an individual can hide their symptoms. But during ‘passing’, they are continuously under fear of getting their stigmatized condition exposed at any time. However, when the stigmatized person becomes discredited, they face other problem such as stigma in the form of avoidance, rejection, discrimination and violence (Goffman, 1963, p. 59). Person (2004) points out that the stigmatizations associated with discrimination often had social ramifications, which then intensify the internalization of stigmatization(Person, 2004,p.359)

The process of stigmatization is facilitated through stigma symbols. Goffman (1963) defines stigma symbols as those signs which convey social information about the stigmatized status of the person. As in Coronavirus cases, forceful home quarantine and the act of pasting stickers with the name of the COVID patient outside the house to make identification of diseased person easier. It highlights the victim’s identity and provides labels such as ‘diseased’ and ‘contagious’ to the person. Thus, these stickers represent stigma symbols providing the label of ‘Coronavirus positive’ to the family and caution others for their safety and hence facilitate the process of stigmatization of family. Moreover, the incidences of tearing those stickers by the stigmatized family (IANS, 2020) highlight the dilemma of COVID patient regarding the disclosure of the social information of their stigmatized status. In Goffman words,

‘*The cooperation of a stigmatized person with normals in acting as his known differentness were irrelevant and not attended to is one main possibility in the life of such a person. However, when his differentness is not immediately apparent, and is not known beforehand, when in fact he is a discreditable, not a discredited, person, then the second main possibility in his life is to be found. The issue is not that of managing tension generated during social contacts, but rather that of managing information about his failing. To display or not to display; to tell or not to tell; to let on or not to let on; to lie or not to lie; in each case, to whom, how, when and where’* (Goffman, 1963, p. 57).

The stigma attached to infectious diseases not only victimised the patient, but it also stigmatized all those who are in close association with the patient, such as partners, family members, and even healthcare providers. As the disease is contagious, so ‘normal’ people fear that these other people can act as carriers of the virus. Goffman (1963) termed this stigma as ‘courtesy stigma’. Thus, this secondary form of stigma creates hurdles for those who are subjected to it. Recent cases in India, where landlords threaten doctors and nurses to evict their residence due to fear of contagious disease, were an explicit example of the same (Jagannath, 2020).

As stigma related to infectious disease creates more adversity, it is crucial to understand the role of stigma in determining the health-seeking behaviour among COVID patients. The anxiety and fear that are now deeply rooted in people's mindset have developed certain negative assumptions among them. Bear (2020) argues that due to lack of sufficient information regarding the process of hospitalization, along with restriction related to visiting the patient in the hospital, had led to confusion and problem in adapting these newly form rules (Bear et al.,2020 p. 6). Moreover, due to the fear of potential stigmatization and social marginalization resulting from the outbreak of any diseases may lead people to deny any clinical symptoms and failure to seek any immediate medical care.

On the one hand, some studies point towards the positive aspect of stigma in infectious disease as it facilitates the avoidance of diseases along with improving personal hygiene. On the other hand, some studies highlight the fact that stigmatization along with discrimination becomes barriers in accessing health care (Fischer et al., 2019; Oaten et al., 2011). There could be severe health problems and difficulty in controlling infectious disease due to those potential barriers. Fischer et al., (2019) argues that during the outbreak of any contagious disease, people are forced to follow the specific guideline to monitor and control the spread of the diseases. Generally, public health officials recommend doing regular testing, taking medication and adopting specific behaviour to prevent infection(Fischer et al., 2019, p.989) During the earlier outbreaks such as HIV, Ebola, and Anthrax, individuals were needed to be identified and to be monitored, and crucial measures were taken to avoid the spread of the virus. But stigma deters these expected behaviours of patients. Studies show that due to health-related stigma and perception, it leads to never receiving any HIV test among black/African American and Hispanic. Along with lower medication adherence and a higher level of depression, anxiety and suicidal ideation. Even stigma related to tuberculosis had also impacted in the practice of contact tracing in outbreak investigation (Fischer et al., 2019, p. 991).

Thus, stigma along with the trust deficit in the public health due to unavailability of vaccination and increasing mortality rate puts people in bewildered condition. Consequently, many cases go unnoticed, which further hinder the breaking of the virus chain. Escaping of people from the quarantine centre highlights this trust deficit. Giridhara Babu, Head-life course Epidemiology at the Public Health Foundation of India, argues that

*‘Escaping quarantine is mostly out of fear and stigma and wanting to be with one’s family since it is for a prolonged time as well as lack of income. It is difficult for people in India to understand the importance of isolation and quarantine, even though it is a response to a pandemic. The faith in the public health system cannot emerge immediately as a response to the pandemic’* (Chetterje, 2020,p.2).

Therefore, due to higher out-of-pocket expenditure for the treatment and the absence of any effective therapy, the pandemic has generated a profound sense of public alarm. It seems to threaten the very survival of the societies in which it has emerged. Hence people are using stigma as a means to avoid these contagious diseases that have a positive aspect in terms of protecting oneself. But, on the other hand, those who are stigmatized are facing many challenges, and they are creating a hindrance in the contact tracing investigation of the COVID patients. Health care personnel are working tremendously with bare minimum resources allocated to them. Still, it is due to the government's failure in controlling this pandemic that India today stands at the 2^nd^ place among other infected countries. Large numbers of dead bodies are piled up in morgues and hospitals. Hence it is crucial to understand how this pandemic is altering the death rituals in India.

Coronavirus pandemic is escalating at a higher pace in India, with a total of 1,11,92,118 cases and 1,57,694 death as on March 06 2021. People are updated daily about these cases through media. It is pivotal to maintain respect for people’s dignity and humanity as it is not just about approximate mathematical figures but living people who are affected by the pandemic. Pathak (2020) has rightly pointed out that this pandemic has made death a mere statistical abstraction; faceless, anonymous and devoid of meaning. The pandemic has seized the opportunity of having dignified last rites from several people. As dignity and respect are two essential aspects attributed to death across all culture, these should not be violated under any circumstance. Even the Supreme Court of India has recommended the government for conducting proper disposal of unclaimed dead bodies found in public places. Even bodies of war victims should be provided dignity. This emphasises the fact that a ‘dead person must be given some respect’ irrespective of circumstances in which the death has occurred (Prajapati & Bhaduri, 2019, p. 57). To understand how COVID 19 is intervening with death rituals allocated within Hinduism, it is essential to have a brief account of those rituals in India

## Sociological Understanding of Death

Generally, there are different understandings of death across cultures and, rites and customs pertaining to death are culture-specific. The criteria by which people define any death as ‘good death’ or ‘bad death’ differs accordingly. Parry (1994) mentions that in a Hindu society, any death is believed to be good if it takes place on a purified ground, and cremation of the body is done in the open air near the bank of a river. It is a held belief that if a dying man hears or chants the name of God at the time of his death, it is considered to be a ‘good death’ in the Hindu culture. Whereas a ‘bad death’ is commonly expressed as ‘untimely death’ in which the deceased couldn’t prepare oneself for dying, such as death by violence, accident or chronic illness. He adds further that in Hinduism, ‘good death is considered a sacrificial act which regenerates the deceased, time, and the cosmos’ (Parry, 1994).

Death is often understood as a physiological demise of an individual. But, Hertz (1960) elaborates on the fact that death does have a specific meaning in the collective consciousness. Society as a whole is involved in certain moral and social obligations which are specific to one culture and hence determined by it (Hertz, 1960, p. 28). Therefore, the death of an individual not only marks the end of the physical body of the individual but it also destroys the social being grafted upon that individual by the society, and to whom the collective representation has attributed importance along with dignity (Hertz, 1960, p. 77).

Every society has specific ritual beliefs and customs regarding funeral rites that vary across culture. While elaborating about the procedure that Hindus follow after the death of their loved ones, Parry (1994) states that: ‘When a person expires, the corpse must be washed, anointed with ghee (clarified butter), wrapped in a white cloth, perfumed and decked with garlands. A piece of gold should be placed into the mouth and nostrils as a means through which the body may be worshipped. The worship of the deceased is called ‘*shava pujan’* in Banaras, where being cremated is believed to end the cycle of incarnation’ (Parry, 1994, p. 179). After mandatory preparation of the dead body is over, people are given a chance to pay last respect and hence mark the beginning of the mourning phase.

Van Gennep (1991) understand mourning as a transitional phase in which mourners enter through rites of separation and then come out of them and remerge in the society through the rites of integration. Interestingly, in some cases, ‘the transitional period of deceased is the counterpart of the transitional period of mourners’ (Robben, 1991, p. 213). Thus, when the deceased soul is believed to get incorporated into the ancestors simultaneously, the mourner is again incorporated back into society.

Hence among the Hindu society, the purpose of the rituals, which are performed in the first ten days after death, is to reconstruct a body for the ‘ethereal spirit’ and provide it with a new form that is believed to be less ‘gross’ than the one the deceased had formerly inhabited(Parry, 1980, p. 89). Thus, it enables the deceased to join his ancestors. Parry (1980) elaborates further that failure to perform theses mortuary rituals adequately might hinder the assimilation of the soul with its ancestors, and it can get stuck in limbo and wanders like a ghost and thus become a constant menace to surviving kin (Parry, 1980, p. 91).

## Significance of Cremation

In Hinduism, cremation entails engaging with the philosophical and mystical dimension of death. Even at an esoteric level in Hinduism, people hold a different view about the time when death is believed to occur. Parry (1994) argues that generally, it is the cessation of physiological functioning of one’s body. But it is believed that vital breath is released by the heat of pyre and ritual of ‘*kapal kriya’* (breaking of one skull). Hence, according to beliefs, death takes place during the process of cremation. Even the smoke from the pyre towards the sky is seen as a metaphor of release of soul and its integration with heaven is considered as ‘good death’ (Davies, 2005, p. 213).

Parry (1994) mentions that, for Hindus, cremation is a sacrifice. The learned Hindus call it ‘*antitiesthi’,* literally ‘the last sacrifice’. It is a sacrificial offering of one’s own self to the gods (Das, 1976, p. 256; Parry, 1994, p. 32).

Further, Das (1976) points out certain commonality in the procedure carried out as a part of cremation and other sacrificial procedures, such as purification of a site, prescriptive use of ritually pure wood, an establishment of ‘*agni’*(fire) with proper use of mantras. The dead body is prepared similarly as the victim of sacrifice and attributed with divinity (Das, 1976, p. 258).

## COVID-19 Compromising With Death Rituals

As there is a higher death rate due to the pandemic, the management of dead bodies and administering of the appropriate last rites to the deceased is a source of concern across countries. Generally, all those deaths in COVID times can be classified as ‘bad deaths’ as they are occurring untimely with people being unprepared for it. Many people are dying desolated and alone in hospitals and at home. Rites and rituals related to death play a crucial role in alleviating the pain of the bereaved family and hence facilitate the grieving process. As there are some held belief of the community associated with those rituals such as the incorporation of the soul of the deceased with their ancestors it is important that rituals be performed effectively (Parry, 1980). But this pandemic has turned the whole world into meek spectators as people just watch those piles of dead bodies waiting for the funeral. Countries have passed on mandatory guidelines and strict rules to control the pandemic, hence forbidding people's movement from one place to another. Bereaved family members are already devastated and shocked due to the sudden death of their loved ones. Added to it, they are obliged to follow those guidelines in a perplexed state of mind. Moreover, due to the irresponsible behaviour of hospital staff, many bereaved families fail to get back the dead bodies of their loved ones (Srivastava, 2020). These all situations lead to distress and anxiety among bereaved families. Though all the rituals mentioned above could not be performed due to the critical condition, at least those dead bodies deserve some respect and dignity.

The Varanasi city celebrates death, and it is believed that whoever dies there attains ‘*Moksha’* (Salvation) (Kaushik, 1976: Parry, 1994). An electrical crematorium was built there in 1991 at Harischandra ghat. Though it has been changed to a gas crematorium, but most of the dead bodies are still cremated manually. The main reason for this is the held belief that manual cremation performed with all rites and rituals facilitates the deceased soul to get salvation. At the same time, a shift from traditional cremation to an electrical crematorium repudiates the performance of certain crucial rituals such as ‘*Parikrama’* (circumambulating around the pyre), ‘*Mukh agn*i(putting fire into the mouth of the deceased) and ‘*Kapalkriya’* (cracking skull of the deceased). These rituals have strong belief and value associated with them. Even the quantity and quality of wood used in a funeral have their significance in claiming one's higher status. Performing rituals are also crucial for binding Hindu society together.

Moreover, immersing ashes into the flowing river is another important aspect of a Hindu funeral. As Hertz (1960) points out that cremation itself is not the final act; it is necessary to perform the complementary rite along with it. As in Indian ritual, after the bodies are burned completely, their ashes should be collected and immersed in the river (Hertz, 1960, p. 43). Parry (1981) elaborates it further and points out that for re-creation of the world, its annihilation is necessary through fire and flood. In the same way, first the deceased body is cremated. Then his ashes are immersed in water to restore it to life (Parry, 1981, p. 267). Thus, immersion of ashes is an important ritual in Hindu society, but people complain that in the electrical crematorium, ashes of persons from different castes get mixed up (Prajapati & Bhaduri, 2019, p. 59). Thus, there are challenges in performing rituals in the electrical crematorium, due to which families choose manual cremation.

But this pandemic has seized this choice from people, and following the WHO guidelines, Indian authorities has instructed using only electrical and gas crematorium (WHO, 2020a). Hence the Coronavirus pandemic has hindered many of the above-mentioned rituals, such as ‘*kapal kriya*’ (breaking of the skull), without which the soul is believed to be trapped within the dead body. Moreover, there are hindrances in the ritual of immersing the deceased's ashes into the flowing river due to the country lockdown. This constraint situation of lockdown and pandemic deters people in performing these rituals, and creating anxiety among bereaved families. Further, increased mortality and inadequate capacity of mortuary services worldwide have created more anxiety as number of dead bodies often surpasses the capacity of the morgue, crematorium and burial sites. In Italy, when the government could not control the spike in dead bodies, the army was called to dispose of those bodies. In Spain, due to a shortage of coffins, parking places in Barcelona were converted into huge makeshift morgues. Condition in Brazil, too, was no different as gravediggers dug mass graveyards. In Spain, the bodies of the elderly were left in the nursing home till the army came to help with the bodies. There were unclaimed dead bodies which lay on the street of Ecuador waiting for the last funeral as the death toll had outpaced the country’s ability to handle (Kumari, 2020). Similar conditions prevail in India, too, as due to the increasing number of dead bodies, hospital staffs are involved in the mass burial of those bodies.

Along with these, countries have passed on strict guidelines and mandatory measures to control the pandemic outbreak, which have impact upon the well-being of bereaved family members. Some countries had banned funeral ceremonies completely, such as China, Ghana, Brazil, and Ecuador. Moore et al. (2020) points out that gatherings have been prohibited altogether in some case, and these practices can have a psychosocial impact on the bereaved families (Moore et al., 2020). Many countries followed certain strict practices, such as discouraging funeral, whereas other countries were forcefully applying forced cremation across all religion. This forceful order to change established funeral practices has led to elicit community response as happened in India too when Brihanmumbai Municipal Corporation (BMC) had ordered that Coronavirus patient bodies would be cremated irrespective of religion (Singh, 2020). Families must be allowed to honour their loved ones by following culture-specific customs to avoid complex grieving. Bear (2020) points out that enforcement of cremation would lead to social disturbance. He recommends that these enforced cremations, especially mass cremation and the delay in releasing dead bodies, should be avoided as there is a high level of anxiety about these possibilities across the communities (Bear et al., 2020, p. 4). Secondly, many countries such as France, South Korea and others, including India, had put a cap on the total number of people who can attend the funeral ceremony along with many rules and guidelines such as maintenance of physical distance during a funeral for all people. Due to the allotment of a fixed number, many mourners were omitted from accompanying the deceased in their last rites.

Another guideline, such as approaching authorities before the performance of funeral rites, leads to delay in these rites. This process of waiting for approval from the authorities created further disturbance and trauma. A reported incidence highlighted that a dead body had to be preserved for two days by the family members due to delay in getting the COVID test report. Doctors had refused to provide the death certificate in the absence of a COVID test report. Its further hampers in providing funeral as mortuaries were not accepting the body in the absence of a death certificate (Banerjee, 2020).

Moreover, hospital staff were seen dragging and dumping dead bodies to a pit for mass burial (Kattimani, 2020). Even many bereaved families struggled to provide a dignified funeral as funeral workers had denied providing cremation or burial to Coronavirus dead bodies due to fear of getting infected (Bhalerao, 2020). Thus, there are significant guilt experiences among the bereaved family members, resulting in poor mental health. (Bear et al., 2020, p. 8).

These incidences highlight that mourning which was understood as a collective representation of the whole society, has been forced to be performed at an individual level due to this disease outbreak. Hence, Moore (2020) recommends that authorities should seek the opinion of the religious head or community members in planning alternatives practices and to fully explain modification in rites along with reason as to why those are required. As evidence from the previous pandemic has suggested that people are willing to adopt new practices about funeral if ‘(i) the new practices meet the symbolic, social and emotional needs of the original ceremonies and practices, and (ii) affected communities themselves are involved in the formulation of any proposed changes’(Moore et al., 2020).

Moreover, there is no denying that funeral workers are under tremendous pressure to provide the funeral to these dead bodies. As the immediate risk of contaminations of Coronavirus from dead bodies to health professional is quite unlikely yet certain guidelines for handling the infected dead bodies are issued by WHO and the Ministry of Health and Family Welfare in India. It recommends leak-proof plastic body bag with a thickness of not less than 150mm and decontaminations of exterior body bags (Government of India, 2020; WHO, 2020b). Further, the WHO had recommended that the dead body should not be washed or embalmed (WHO, 2020b). As Coronavirus is highly contagious so people who deal with dead bodies are at higher risk. thus, it is essential that precautions are maintained. WHO had recommended that only the trained staffs wearing PPE Kits should be allowed for the disposal of the dead bodies following all the preventive measures. Further, for proper disposal of bodies to be cremated, the Indian Council of Medical Research had preferred using electrical or compressed natural gas crematorium (Vidua et al., 2020). But the way India is handling this immediate threat is tenuous. This raises a pertinent question on the role of the authority in effective planning as well as their dealing mechanism with the pandemic.

As mentioned above in Hinduism, people often prefer traditional cremation rather than electrical/gas crematorium. But most of the time, there are technical snags in the functioning of the electrical crematorium too. For instance, in Varanasi, the gas crematorium present at Harischandra ghat remains closed for several months. Even the crematorium's capacity is less as there are only two chambers to cremate bodies (Kumari, 2020). In this perilous time, the government doesn’t seem prepared enough to stop these issues of technical snag, as during the peak of COVID-19, the gas-fueled incinerators of the electrical crematorium of Nigambodh Ghat in Delhi broke down. As a result, dead bodies were sent back to morgues in hospitals, where already piles of dead bodies were waiting for the past five days to be cremated (Kumar, 2020a). As one of the officials in his interview to Hindustan times states that: ‘*the backlog of such cases is increasing every day. Wearing PPE suits, we stand in the sun in this heat outside the crematorium only to be told in the evening that they cannot accept the bodies. Today there are 28 bodies on the floor lying next to each other or piled up on top of each other. Last week, there were 34*’(ibid).

Due to this scenario, immediate orders were passed to cremate bodies on a wooden pyre, which goes against guidelines given by WHO. As a concern for their safety, staff members of the crematorium had threatened to quit the job. Similarly, the crematorium in Ghaziabad had stopped working with the half-burnt body of COVID 19 patient inside it, and it took around 29 hours to repair the same (Ghosh, 2020). During that time in Varanasi too, the gas crematorium stopped functioning for around four to five days due to technical snag. Due to this, authorities were pressuring Dom^1^ community to burn those body on the wood pyre. Dom community were tensed as authorities had not provided them with any PPE kits, nor given any training about the mechanism of prevention from the infectious disease^2^. In normal circumstances, too, Dom doesn’t have any safety equipment to burn dead bodies. As Kumari and Guite (2019) points out that they use a small bamboo shaft and a piece of cloth to burn the body and protect themselves against scorching heat and smoke. During monsoon season situation turns more complicated as the level of Ganges water reaches up to the street. Due to this, the electrical crematorium remains dysfunctional for several months. As cremation ghat too gets submerged in water, because of space constraints, the Dom community cremates those bodies in their locality (Kumari & Guite, 2019, p. 254). But during monsoon season of 2020, there grew more anxiety and fear due to the highly infectious nature of Coronavirus. As crematorium had stopped functioning and the water level had increased. Thus, PPE kits were mandatory for them, for which they were raising their demand in front of higher authorities. But wearing PPE kits while burning a body can lead to accidents as plastic can melt due to higher temperature rising from the pyre.

Moreover, there were reports of insufficient material resources for a proper funeral in India due to the country's sudden lockdown as cremations were facing challenges in getting enough wood supply (Amrita, 2020). Thus, these incidences highlight the ways in which India is dealing with this pandemic.

## Conclusion

Infectious diseases are stigmatized due to its contagious nature and the unavailability of any cure further facilitates the stigmatization of the infected individual. This stigmatization hinders in health-seeking behaviour of people and affects the process of contact-tracing too. The government has taken many guidelines to control the pandemic. But these measures only deny the right of an individual to be with his family members in their last days, due to which people are forced to die in isolation. People are not allowed to kiss or hug the deceased and thereby give a vent to their emotions. All these situations lead to tremendous anxiety and stress among the deceased families. Molokhia and Waqas (2020) argues that the death of family members is identified as a risk factor that can lead to any post-traumatic stress disorders in family members such as anxiety, panic disorder and depression (Molokhia & Waqas  2020,p1**).** Though immediate and strict measures are required to control the pandemic, still authorities should take the bereaved families into considerations. Despite the fact that the pressure on the frontline workers is immense, incidences like the missing of dead bodies from the hospital and dumping and dragging of dead bodies for mass burial amounting to disrespecting the deceased cannot not be justified on any ground.

## Notes

1 Dom community are engaged in caste-based occupation of burning the dead bodies.

2 Informed by telephonic conversation.
